# Epidemiological and Clinical Characteristics of Acute Dengue Virus Infections Detected through Acute Febrile Illness Surveillance, Belize 2020

**DOI:** 10.3390/v14040768

**Published:** 2022-04-07

**Authors:** Anh N. Ly, Russell Manzanero, Adrianna Maliga, Sarah M. Gunter, Shannon E. Ronca, Emily Zielinski-Gutierrez, Francis Morey, Kim Bautista, Andres Espinosa-Bode, Beatriz López, Loren Cadena, Rafael C. Fuentes, Timothy A. Erickson, Flor M. Munoz, Joy Mackey, Gerhaldine Morazán, Kristy O. Murray

**Affiliations:** 1 Department of Pediatrics, National School of Tropical Medicine, Baylor College of Medicine and Texas Children’s Hospital, Houston, TX 77030, USA; anh.ly@bcm.edu (A.N.L.); adrianna.maliga@bcm.edu (A.M.); sm22@bcm.edu (S.M.G.); ronca@bcm.edu (S.E.R.); timothy.erickson@bcm.edu (T.A.E.); florm@bcm.edu (F.M.M.); gerhaldine.morazan@bcm.edu (G.M.); 2 Department of Epidemiology and Environmental Sciences, The University of Texas Health Science Center at Houston, Houston, TX 77030, USA; 3 Belize Ministry of Health and Wellness, Belmopan, Belize; russell.manzanero@bcm.edu (R.M.); fmorey@health.gov.bz (F.M.); kbautista@health.gov.bz (K.B.); 4 The William T. Shearer Center for Human Immunobiology, Texas Children’s Hospital, Houston, TX 77030, USA; 5 Division of Global Health Protection, Center for Global Health, Centers for Disease Control and Prevention—Central America Region, Guatemala City 01015, Guatemala; ebz0@cdc.gov (E.Z.-G.); htx9@cdc.gov (A.E.-B.); fdx8@cdc.gov (B.L.); bqp3@cdc.gov (L.C.); qin8@cdc.gov (R.C.F.); 6 Department of Emergency Medicine, Baylor College of Medicine, Houston, TX 77030, USA; joy.mackey@bcm.edu; 7 Central Medical Laboratory, Belize Ministry of Health and Wellness, Belize City, Belize

**Keywords:** dengue virus, surveillance, acute febrile illness, Central America, Belize

## Abstract

The Acute Febrile Illness (AFI) Surveillance Network in Belize is a country-wide active surveillance program aimed at diagnosing vector-borne, respiratory, and enteric pathogens among patients presenting to 11 participating hospitals and clinics with new onset fever. This study describes the epidemiology of dengue virus (DENV) infections in Belize diagnosed through AFI surveillance in 2020. Of the 894 patients enrolled and PCR-tested for DENV in this period, 44 DENV-positive cases (5%) were identified. All four DENV serotypes were detected, with two cases testing positive for DENV serotype 4, which is the first report of this serotype in Belize since 2004. The majority of DENV cases (66%) were diagnosed in the Belize District, which contains the largest urban center in the country (Belize City). Positive cases were detected between January 2020 and September 2020, with the majority (89%) diagnosed during the dry season between January and April, unlike years prior when cases were more often diagnosed during the wet season. Clinical signs and symptoms varied slightly between DENV serotypes. Active surveillance of DENV among AFI cases provides insight into the epidemiologic and clinical characteristics of DENV in Belize. This information is important for informing public health interventions to mitigate DENV transmission.

## 1. Introduction

Dengue virus (DENV) is a mosquito-borne positive sense single-stranded RNA virus in the flavivirus family. This neglected tropical disease (NTD), as defined by the World Health Organization (WHO), is a significant global health threat, with approximately three billion people around the world at high-risk for infections in mostly low to middle income countries (LMIC) [1,2]. While DENV infection is rarely fatal with adequate case management, it is a significant source of morbidity in tropical and sub-tropical regions resulting in a large loss in disability adjusted life years, especially compared to other NTDs [3]. Over the last two decades, the number of dengue cases reported worldwide have increased over eight fold, from >500,000 cases in 2000 to 4.2 million cases in 2019 [4]. The rise in DENV infections can be attributed to large-scale factors such as population size, urbanization, travel, climate change, and poor sanitation, in addition to limited resources for diagnostics and vector control [5].

Belize is among the countries in Central America at risk of DENV outbreaks. Belize is a relatively small country with an estimated population of 419,000 and is geographically slightly smaller than the U.S. state of Massachusetts [6,7]. Bounded by Mexico, Guatemala and the Caribbean, Belize has a tropical climate with temperature ranging from 17.7 °C to 31.3 °C [8]. The country is classified as an LMIC with high rates of poverty, specifically concentrated in rural regions [9,10]. The main vector species for DENV, *Aedes aegypti* and *Aedes albopictus*, are naturally occurring throughout the country. The combination of tropical climate, limited resources, and pockets of extreme poverty may put this population at increased risk for DENV transmission. Specifically, communities with high rates of poverty may be exposed to additional environmental risks such as limited sanitation services and poor-quality housing, emphasizing the need for prevention and control of vector-borne diseases, such as dengue, to reduce morbidity among these vulnerable populations.

Despite the documented ongoing transmission of DENV in Belize [10], our understanding of the epidemiology in the country remains limited. Prior epidemiologic investigations were limited to serologic surveillance with a small sample size, which limit our ability to determine transmission dynamics [11]. Active surveillance targeting acute cases is critical for timely implementation of effective public health messaging and vector control measures to eliminate disease transmission. The Belize Acute Febrile Illness (AFI) Surveillance Network, launched country-wide in January 2020, was designed specifically to provide diagnostic infrastructure to determine acute infections in near real time from endemic and emerging pathogens, such as DENV [12]. To our knowledge, the current report will be the first known to provide a comprehensive epidemiological understanding of DENV in Belize through an active surveillance approach. Herein, we present the findings of the epidemiology of DENV infection in Belize identified through AFI Surveillance Network in Belize in 2020. 

## 2. Materials and Methods

### 2.1. Study Population

Patients were recruited through the AFI Surveillance Network, a multisite project in collaboration between the Belize Ministry of Health and Wellness (MOHW), Baylor College of Medicine, and the U.S. Centers for Disease Control and Prevention (CDC). The surveillance system covers all six districts of Belize with active enrollment at 11 major MOHW-run clinics and hospitals. Any patients 60 days or older presenting with fever of ≥100.4 °F (38 °C) or having had a history of fever within the past 7 days were invited to participate in the study, regardless of gender, race, or ethnicity. Patients presenting to the facility with a known cause of febrile illness or clear cause of infection were excluded (i.e., wound abscess). In response to the COVID-19 pandemic, on 13 April 2020, we expanded the inclusion criteria to include patients reporting two or more acute onset events of respiratory or gastrointestinal symptoms in the absence of fever. Each eligible patient was consented and had blood specimens and relevant demographics, clinical, and epidemiological data collected. This study focuses on patients presenting for medical care between 1 January 2020 and 31 December 2020. This study was approved by the Baylor College of Medicine IRB (Protocol H-44070) and the CDC (0900f3eb819aa27b).

### 2.2. Data Collection and Laboratory Testing

Enrolled patients were interviewed during the clinic visit to gather their demographic information, recent travel history (domestic or international), animal exposure, vector exposure, and clinical presentation. The interviews were conducted in person and survey data were entered into REDCap. Clinical records on positive cases were abstracted to capture data on clinical course and laboratory findings. We then determined if any positive cases met the WHO case definition of fever and two or more of the following signs and symptoms: nausea, vomiting, rash, arthralgia, headache, retroorbital pain, muscle pain, chest pain, leukopenia, abdominal/stomach pain, fatigue, elevated hematocrit with thrombocytopenia [13].

Blood samples were collected from participants to test for vector-borne pathogens, by real-time multiplexed PCR, which tested for Zika virus [14,15], West Nile virus [16], chikungunya virus [17], and DENV [18]. The pan-DENV assay has a sensitivity of 10 copies/reaction. If a patient was identified as DENV positive with the initial pan-DENV test, an additional RT-PCR was performed to determine the specific DENV serotype [19]. All testing was performed in the Central Medical Laboratory in Belize, and positive results were confirmed at Baylor College of Medicine.

### 2.3. Data Analysis 

Statistical analyses were performed using Stata 16.1 (StataCorp LLC, College Station, TX, USA) [20]. Descriptive statistics were calculated for all variables. Bivariate analysis was conducted to determine the odds ratio (OR) of DENV infection for different demographic, risk factor, and presenting symptom groups. Fisher’s exact was used for any variable with ≤5 observations in a category. The temporal distribution of dengue cases by month, distribution of reported symptoms by serotypes, and clinical outcomes were also analyzed. The continuous variable “age” was converted into a categorical variable for analysis. Since the median age among DENV-positive cases was 21, the age variable was converted to a binary variable with a cutoff at 20 for analysis. Other categorical variables were regrouped as appropriate for analysis. Race and ethnicity were obtained from patient medical records. Records where race, ethnicity, and/or occupation were listed as unknown were recoded as missing. Fisher’s exact *p*-values and confidence intervals were reported for comparisons with cell sizes containing fewer than 5 observations. Bonferroni correction was applied for the multiple comparison of signs and symptoms. A multivariable logistic regression model was built using the backward-stepwise variable selection method. Variables with a *p*-value < 0.25 at the bivariate level were considered for inclusion in the model. The final model was determined by removing variables based on *p*-values until only those with a *p*-value ≤ 0.05 remained. Geospatial mapping was performed on ArcGIS Desktop 10.8 [21]. The shapefile of Belize administrative boundaries was obtained from the Biodiversity and Environmental Resource Data System of Belize [22]. Data from specific health facilities were aggregated to the district level. 

## 3. Results

Between January and December 2020, 944 patients were enrolled through the Belize AFI Surveillance Network. A total of 894 (95%) that had samples available for vector-borne disease testing were included in this analysis. Among the 894 patients, 78% were under the age of 45, with 31% under the age of 15 (Table 1). The proportion of female and male were similar, 53% and 47%, respectively. A high proportion of patients identified as more than one race (28%), followed by Black (24%). Over one-third of the cohort (38%) identified as Hispanic or Latino. Among all six districts, Belize District received the highest number of patients (29%) followed by Toledo District (22%), which is in the southernmost part of the country. 

Among the 894 AFI patients tested for DENV, 44 (5%) tested positive. Of the DENV-positive cases, 61% (27/44) were infected with a single serotype while the other 39% (17/44) were co-infected with multiple serotypes. DENV-1 and DENV-2 were prevalent among mono-infected cases, with 30% (13/44) of positive cases being DENV-1 and another 30% (13/44) being DENV-2. Nearly one-third of the positive cases were co-infected with DENV-1 and DENV-3 (30%; 13/44). DENV-4 was detected among two patients and both cases were co-infected with either DENV-1 or DENV-3. There was one patient who was co-infected with three serotypes (DENV-1/DENV-3/DENV-4). 

The median age for DENV-positive cases was 21 years of age compared to 27 for DENV-negative cases (Table 1). An equal number of DENV-positive patients were under and over 20 years of age, while a majority (63%) of DENV-negative cases were ≥20 years old (Table 2). No positive dengue cases were under a year of age. The proportions of DENV-positive cases between females and males were the same (50% each). While the distribution of race varied more widely among DENV-negative patients, most DENV-positive patients were Black or identified as more than one race (23% and 27%, respectively) (Table 1). 

Most (89%) DENV-positive patients sought care in the northern districts of Belize (Belize District, Corozal District, and Orange Walk District) compared to only 46% of DENV-negative patients. Indoor occupation was similar among DENV-positive patients (86%) and DENV-negative patients (80%) (Table 1).

Table 2 describes the distribution of participants by demographic and epidemiologic factors and the bivariate relationship between these factors and risk of DENV infection. Over three-quarters (77%) of DENV-positive patients reported contact with mosquitos two weeks prior to symptom onset, compared to 52% of those who tested negative for DENV (OR = 3.1, *p*-value = 0.002). Additionally, 89% of positive cases visited a clinical site in the northern region compared to 46% of those who tested negative (OR = 9.1, *p* < 0.001). Among the DENV-positive cases, over one-third (39%) were students compared to 17% of those who tested negative (OR = 3.0, *p* = 0.001). All other variables were not statistically associated with risk for infection. 

Based on results of the bivariate analysis (Table 2), the following variables with *p* ≤ 0.25 were considered for inclusion in the multivariable logistic regression: contact with mosquito, contact with sick individuals, student status, age, race, and region of clinical site. Using a backward stepwise model building approach, we constructed a final model (Table 3). Reporting contact with a mosquito within 2 weeks of symptom onset (OR = 2.8; *p* = 0.007), presenting for medical care at a northern clinical site (OR = 8.3; *p* < 0.001), and being a student (OR = 2.3, *p* = 0.015) were independently associated with DENV infection. 

**Figure 1:**
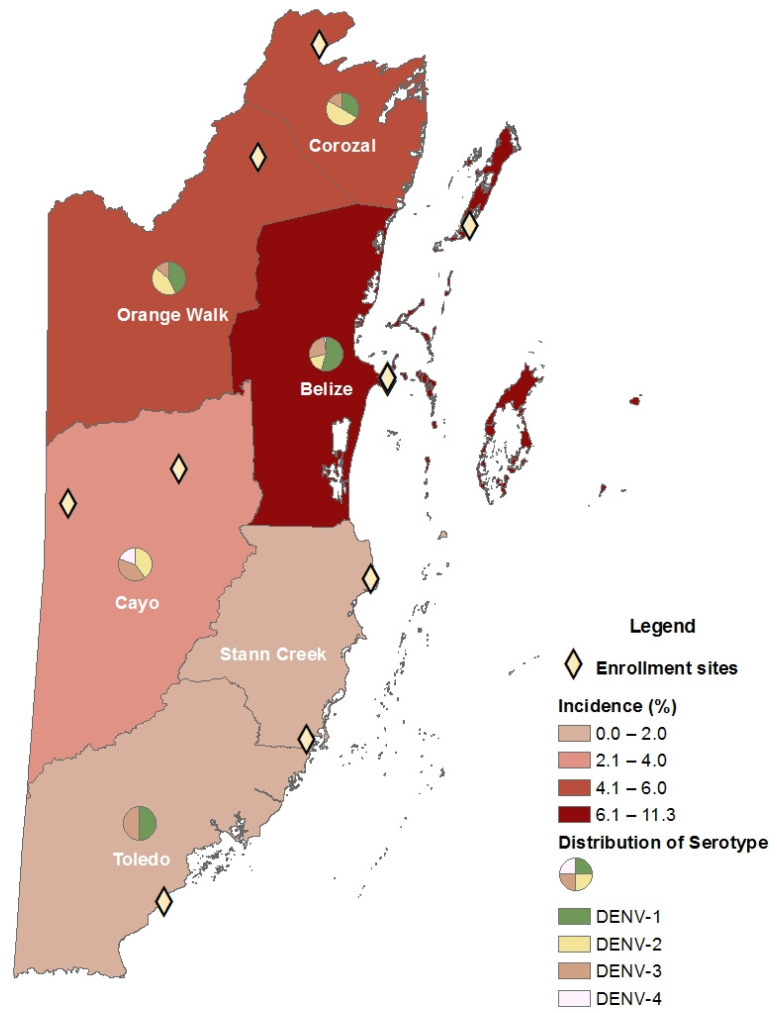
Incidence of DENV infection and distribution DENV serotype by district.

The incidence of infection among individuals enrolled in the AFI Surveillance Network by district and the geographical distribution of serotypes are shown in Table 4 and Figure 1. The highest incidence of infection was observed in northern districts of Belize, Corozal, and Orange Walk. Stann Creek District was the only district that had no DENV-positive cases identified. Belize District was the only district region where all four circulating DENV serotypes were identified.

**Figure 2:**
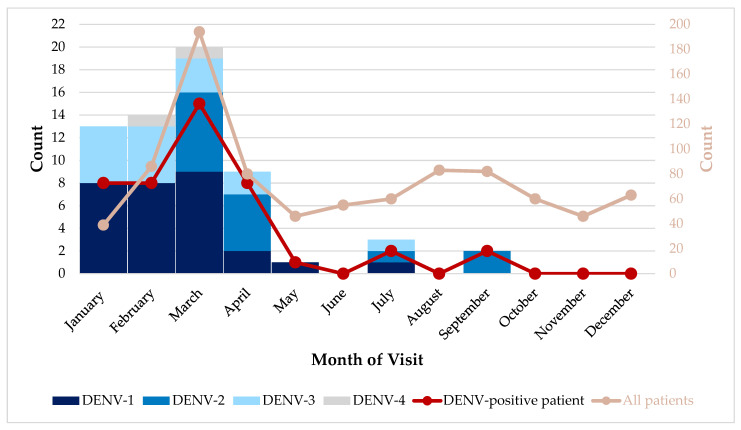
Dates of clinical presentation of DENV-positive patients and all participants (*n* = 894) between 1 January 2020 and 31 December 2020 and distribution of DENV serotype detected by month.

The majority of the DENV-positive cases were identified in the first part of 2020, between January and April (39 of 44; Figure 2). There were no cases identified in June, August, October, November, or December 2020. DENV-1 and DENV-3 were detected in most of the months with positive cases. DENV-2 cases were detected from March 2020 and onward. The DENV-4 cases were identified in February and March 2020. Figure 2 also shows time at which the 894 patients visited an AFI clinical site. 

General, respiratory, and gastrointestinal signs and symptoms reported by patients and the odds of infection associated with presenting symptoms are shown in Table 5. Signs and symptoms were also stratified by DENV serotype. Among the 44 positive cases, fever (98%), headache (59%), chills (43%), muscle pain/aches (36%), retroorbital pain (34%), and arthralgia (32%) were the most common general symptoms reported. Cough (39%), sore throat/painful swallowing (25%), and coryza/congestion/rhinorrhea (23%) were the most common respiratory symptoms among all DENV-positive patients. Fewer positive cases reported gastrointestinal symptoms in comparison, but vomiting (23%), diarrhea (18%), and nausea (18%) were the most common. DENV-positive patients were significantly more likely to report arthralgia, retroorbital pain, muscle pain/aches, chills, and loss of appetite as compared to AFI cases with other diagnoses.

Fever, headache, chills, and cough were commonly reported among DENV-1 (100%, 52%, 38%, and 31%, respectively), DENV-2 (93%, 80%, 53%, and 40%), DENV-3 (100%, 44%, 44%, and 38%, respectively), and DENV-4 cases (100%, 100%, 100%, and 100%, respectively). Retroorbital pain was also common among DENV-1 (38%) and DENV-2 cases (40%) while muscle/pain/aches were common among DENV-2 (47%), DENV-3 (38%), and DENV-4 (100%). Arthralgia was the most common symptom among DENV-2 cases (53%) following fever and headache. High proportions of DENV-3 (38%) and DENV-4 (100%) cases also reported coryza/congestion/rhinorrhea. 

Table 6 displays the clinical outcomes of the DENV-positive cases. Thrombocytopenia and leukopenia were most common (23% each); five DENV-positive cases (11%) had platelet counts <100,000/μL. For both thrombocytopenia and leukopenia, eight of ten cases were infected with a single DENV serotype. Two DENV-positive cases (5%) were hospitalized for 2 days and 4 days, respectively, both of whom were infected with DENV-2. There were no deaths among the DENV-positive cases. 

Of the 44 positive cases, 25 (57%) met the WHO suggested case definition for dengue with or without warning signs. One 29 year-old case who was hospitalized for 4 days had evidence of warning signs with thrombocytopenia (platelets 41,000/μL), elevated liver enzymes (AST = 335 U/L and ALT = 235 U/L), vomiting, and vomiting/abdominal pain. The other hospitalized case had thrombocytopenia (73,000/μL) and vomiting; however, liver enzymes were normal, and the patient did not report abdominal pain. One non-hospitalized case had some evidence of warning signs with vomiting, abdominal pain, and thrombocytopenia. None had evidence of elevated hematocrits, plasma leakage or met the criteria for severe dengue [13]. 

## 4. Discussion

Over the course of one year of country-wide acute febrile illness surveillance in Belize, we identified 44 patients presenting with acute DENV infection, representing 5% of the overall cohort of care-seeking febrile patients. We detected all four DENV serotypes in 2020, including the first case of DENV-4 reported from Belize in 16 years [24]. DENV-4 may have been circulating in Belize but could have escaped detection because of passive surveillance and limited resources for diagnostic testing and serotyping. DENV-4 has the lowest number of reported cases worldwide compared to the other DENV serotypes [25]. Our surveillance data showed that DENV-1 and DENV-2 were the dominant serotypes among the mono-infected cases. The detection of DENV-2 is of public health significance because this serotype is associated with greater clinical severity and higher mortality rates compared to other serotypes [25]. Consistent with this finding, both of our hospitalized DENV-positive cases were infected with DENV-2, supporting greater clinical severity of this serotype. In 2019, PAHO also expressed concerns about DENV-2 affecting children and adolescents [26]. Our investigation identified co-infection in 39% (17/44) of DENV-positive cases, with co-infection of DENV-1 and DENV-3 most common (13/17, 76.5%). In endemic regions like Belize, co-infections with multiple serotypes are possible, ranging from 5.5% to 42.9% of infections [27,28,29,30,31,32]. The presence of multiple DENV serotypes increases the risk of severe dengue, including dengue hemorrhagic fever and dengue shock syndrome, due to secondary infections [4,33]. However, in our study, there were more severe clinical outcomes among those that were infected with one DENV serotype (serotype 2) compared to those who were concurrently infected with two or more serotypes. 

There was an overall higher incidence of acute DENV infection and more diverse serotype circulation in the northern half of Belize. The incidence of DENV infection in Belize District was more than two times the incidence observed among AFI participants among other districts in the study. Our population-based incidence also showed higher burden of disease in northern districts of the country compared to southern districts. Our finding of high burden of disease in Belize District, where the most populous city, Belize City, is located, is consistent with the Ministry of Health and Wellness’s report of DENV infections in urban areas being threefold those of rural areas [10]. The second largest urban center in Belize is Belmopan, the capital city located in Cayo District; however, only 9% of dengue cases we observed were reported from that district.

There are multiple factors that could explain the higher incidence in Belize District. Belize District contains major urban centers including Belize City and San Pedro. The district is also a centralized location with popular tourist destinations and major seaports [7,34]. The constant movement of people in and out of these urban areas facilitate opportunities for transmission of infectious pathogens and may also explain our ability to identify all four DENV serotypes in Belize District. Furthermore, *Aedes* spp. mosquitoes are well adapted to urban settings [4,35,36]. The higher instance of DENV infection in the northern districts could also be explained by unique ecologic characteristics. The southern part of the country consists of mountainous regions with pine and broad-leaved forest. By contrast, the northern part of the country is lowland consisting of more savanna, broad-leaf forest, agricultural use land, and wetland. The ecologic features of the northern portion of the country may be better suited for *Aedes* spp. mosquito breeding. 

The age distribution for our patient population is close to the estimated Belize population in 2020 [6]. The median age of DENV-positive patients was lower when compared to DENV-negative cases, while the finding was not significant (*p* = 0.08) it is consistent with previous reports [26,37]. One reason for a higher proportion of infections among younger populations may be due to the lack of immunity because of less time for the opportunity of exposure to the virus compared to older adults [26]. Even with immunity to a specific serotype, children can still be infected with a different DENV serotype, especially with the co-circulation of all four serotypes in Belize. The high proportion of infection among children and young adults in Belize emphasizes the importance of vector control, education, and focused surveillance in this group. 

Although all DENV-positive cases were laboratory confirmed with PCR testing, only (25/44) 57% met the WHO suggested case definition of dengue. Although only 25 of the 44 cases presented with fever and two of the mentioned signs or symptoms, eight cases presented with fever and one additional symptom, and another ten cases presented with only fever. There was only one case that presented with one of the mentioned symptoms with no fever. This finding emphasizes the importance of continued acute febrile illness surveillance to enhance the detection and diagnoses of diseases beyond the traditional case definitions. 

Positive cases in our study reported a wide range of clinical signs and symptoms. Some signs and symptoms were found associated with DENV infection in our study, such as arthralgia, retroorbital pain, muscle pain/aches, and headache. These symptoms are consistent with criteria under the WHO suggested case definition of dengue [13]. We also found that chills and loss of appetite were associated with DENV infection. A meta-analysis of global epidemiology outbreaks identified symptoms such as myalgia, chills, and retroorbital pain as significant predictors, all of which were also significant in our study [25]. 

Due to the small sample size, we did not calculate a measure of association for each DENV serotype and clinical signs and symptoms; however, there are certain distinctions in the distribution of signs and symptoms that may still be clinically relevant. Among DENV-1 positive patients, headache was the second most commonly reported symptom, followed by retroorbital pain, chills, and cough. These same signs and symptoms were common among DENV-2, along with arthralgia and muscle pain/aches. For DENV-3 positive patients, headache, muscle pain/aches, chills, and cough were reported frequently, and 38% of the DENV-3 patients also reported coryza/congestion/rhinorrhea. Both of the DENV-4 positive cases presented with fever, headache, muscle pain/aches, chills, cough, and coryza/congestion/rhinorrhea. 

We observed a decline in DENV-positive cases after March 2020 of our study period, with few to no cases detected during the second half of 2020. All positive dengue cases identified in our study were reported to the Belize Ministry of Health and Wellness Vector Control unit, who then responded with mosquito abatement activities; however, we do not believe this action alone drove the cases down countrywide after March 2020. Historically, Belize experiences the highest rate of DENV infection during the rainy season (June to November) [7,38]. With the 2020 data, we expected the incidence of mosquito-borne infections in Belize to be higher around October to December due to significant flooding caused by three major hurricanes that impacted Belize during the Atlantic Hurricane Season [39,40,41]. While the DENV infection trend we observed was different from the usual seasonal expectation, it is consistent with the 2020 trend reported by PAHO with higher number of cases per week during the first three months of the year [38]. This is most likely related to the COVID-19 pandemic and related control measures [42,43]. The restrictions may have altered community behaviors, such as spending time outdoors, reducing the risk of mosquito contact or altered health seeking behavior as seen with fewer visits to AFI clinical sites overall in the second half of 2020. 

This study has several other limitations worth noting. One important limitation is the low number of DENV-positive cases, which can affect statistical precision. Another limitation is the possibility of selection bias associated with participating clinical sites. Although our clinical sites are representative of every district in Belize, public Ministry of Health and Wellness sites were prioritized for participation. Very few patients (*n* = 89) were enrolled from private hospitals or clinics. In Belize, all citizens have free access to healthcare through the public healthcare system, though those with greater financial means often choose to seek care from private clinics. This could bias the data towards inclusion of those of a lower socioeconomic status. Furthermore, there may be selection bias with the population of participants who chose to enroll in the study compared to those who declined. Unfortunately, we do not have access to the baseline number of patients who would have been eligible for inclusion; therefore, we are unable to measure overall participation rates and characteristics of non-participants. Information such as exposure and risk factors were self-reported by patients; thus, there may be recall bias associated with the information. The estimated incidence by geographical area was based on the location of the clinical facility where the patient presented for care, which may not be in the same district as the patient’s residence. Residential address would be needed to provide a more accurate estimate of incidence by geographical area. Furthermore, since the AFI Surveillance Network requires participant consent, the estimated population incidence may not reflect the true incidence of all cases in the country. Lastly, many DENV-positive cases had no race or ethnicity listed in their medical record, which prohibited us to from being able to categorize them. As such, they were excluded from the analysis, which could impact our findings. 

Despite these limitations, we believe our study presents novel and important findings. Our study was the first active surveillance study in Belize to prospectively identify acute DENV infections and evaluate high-risk groups, risk factors, and clinical signs and symptoms associated with infection. In our collaboration with a network of major hospitals and clinics across all districts of the country, we were able to determine the spatial distribution of DENV infections and DENV serotypes in different regions of Belize. Our surveillance also detected the less prevalent DENV serotype 4, which had escaped detection for 16 years. 

## 5. Conclusions

Our study found that 5% of those presenting with acute febrile illness were positive for DENV infections in Belize. Through our active surveillance approach, we were also able to identify cases of DENV-4 for the first time in almost two decades, and we were able to identify high risk groups, geographic locations, and mosquito exposures as important risk factors for DENV infection. Continued active dengue surveillance in Belize is important to ensure timely outbreak response efforts.

## Figures and Tables

**Table 1:** Characteristics of participants presenting with acute febrile illness in Belize, between 1 January 2020 and 31 December 2020.

Variable	Total	DENV-Positive	DENV-Negative
	*n* = 894	*n* = 44	*n* = 850
	*n* (%)	*n* (%)	*n* (%)
**Age**			
Median age, year (IQR)	27 (33)	21 (22)	27 (34)
Under 15 years	277 (31)	15 (34)	262 (31)
15–29	224 (25)	13 (30)	211 (25)
30–44	196 (22)	10 (23)	186 (22)
45–59	118 (13)	4 (9)	114 (13)
60 or older	74 (8)	2 (5)	72 (8)
**Sex**			
Male	421 (47)	22 (50)	399 (47)
Female	473 (53)	22 (50)	451 (53)
**Indoor Occupations**			
Professional, technical, managerial	166 (19)	5 (11)	161 (19)
Clerical support	6 (1)	0 (0)	6 (1)
Domestic services	51 (6)	6 (14)	45 (5)
Sales and services	74 (8)	4 (9)	70 (8)
Student	163 (18)	17 (39)	146 (17)
Unemployed	107 (12)	3 (7)	104 (12)
Under school-age children	156 (17)	3 (7)	153 (18)
**Outdoor Occupations**			
Agriculture, forestry, fishery	26 (3)	0 (0)	26 (3)
Armed forces	21 (2)	0 (0)	21 (2)
Skilled manual	29 (3)	1 (2)	28 (3)
Unskilled manual	28 (3)	3 (7)	25 (3)
**Race**			
Black	215 (24)	10 (23)	205 (24)
American Indian/Ketchi/Mopan/Mayan	130 (15)	1 (2)	129 (15)
White	92 (10)	2 (5)	90 (11)
Asian	36 (4)	0 (0)	36 (4)
More than one race	253 (28)	12 (27)	241 (28)
Unknown	168 (19)	19 (43)	149 (18)
**Ethnicity**			
Not Hispanic or Latino	389 (44)	11 (25)	378 (44)
Hispanic or Latino	338 (38)	14 (32)	324 (38)
Unknown	167 (19)	19 (43)	148 (17)
**Clinical Site in North Belize**			
Belize	257 (29)	29 (66)	228 (27)
Corozal	67 (7)	4 (9)	63 (7)
Orange Walk	107 (12)	6 (14)	101(12)
**Clinical Site in South Belize**			
Cayo	134 (15)	4 (9)	130 (15)
Stann Creek	134 (15)	0 (0)	134 (16)
Toledo	195 (22)	1 (2)	194 (23)
**DENV mono-infection**			
DENV-1	-	13 (30)	-
DENV-2	-	13 (30)	-
DENV-3	-	1 (2)	-
DENV-4	-	0 (0)	-
**DENV co-infections**			
DENV-1/ DENV-2	-	2 (5)	-
DENV-1/DENV-3	-	13 (30)	-
DENV-1/DENV-3/DENV-4	-	1 (2)	-
DENV-3/DENV-4	-	1 (2)	-

- Not applicable.

**Table 2:** Bivariate relationship between risk factors and DENV infection.

Variable	DENV-Positive *n* = 44 *n* (%)	DENV-Negative *n* = 850 *n* (%)	Odds Ratio (95% CI)	*p*-Value *
Contact with mosquito ^1^	34 (77)	442 (52)	3.1 (1.5, 6.4)	**0.002**
Contact with sick individuals ^1^	10 (23)	119 (14)	1.9 (0.9, 3.9)	**0.10**
Travel history ^2^	6 (14)	95 (11)	1.2 (0.5, 3.0)	0.64
Outdoor occupation ^†^	4 (9)	99 (12)	0.7 (0.2, 2.1)	0.81
Student	17 (39)	146 (17)	3.0 (1.6, 5.7)	**0.001**
Twenty years or older	22 (50)	533 (63)	0.6 (0.3, 1.1)	**0.08**
Female sex	22 (50)	451 (53)	0.9 (0.5, 1.6)	0.70
Black race	10 (23)	205 (24)	1.6 (0.7, 3.6)	**0.25**
Hispanic or Latino	14 (32)	324 (38)	1.5 (0.7, 3.3)	0.34
Clinical site in northern region ^†^	39 (89)	392 (46)	9.1 (3.5, 29.9)	**<0.001**

^1^ Within 2 weeks of symptom onset. ^2^ Within 30 days of symptom onset. ^†^ Fisher’s exact *p*-value and confidence interval reported. * *p*-values in bold were entered into multivariable model.

**Table 3:** Multivariable logistic regression model with final variables identified as independently associated with risk for DENV infection.

Variable	Odds Ratio (95% CI)	*p*-Value
Contact with mosquito ^1^	2.8 (1.3, 5.7)	**0.007**
Clinical site in northern region	8.3 (3.2, 21.5)	**<0.001**
Student	2.3 (1.2, 4.4)	**0.015**

^1^ Within the 2 weeks of symptom onset. *p*-values in bold were entered into multivariable model.

**Table 4:** Incidence of DENV by district.

District	Total Enrolled	Total DENV Positive	Cohort Incidence (%)	Belize Population 2020 [23]	Population Incidence (per 100,000)
Corozal	67	4	6.0	50,490	7.9
Orange Walk	107	6	5.6	53,373	11.2
Belize	257	29	11.3	127,683	22.7
Cayo	134	4	3.0	102,115	3.9
Stann Creek	134	0	0	46,015	0
Toledo	195	1	0.5	39,525	2.5

**Table 5:** Distribution of reported signs and symptoms and odds of DENV infection with specific signs and symptoms.

Sign/Symptom	DENV- Positive *n* = 44 *n* (%)	DENV-Negative *n* = 850 *n* (%)	OR (95% CI)	*p* Value *	DENV-1 *n* = 29 *n* (%)	DENV-2 *n* = 15 *n* (%)	DENV-3 *n* = 16 *n* (%)	DENV-4 *n* = 2 *n* (%)
General								
Fever ^†^	43 (98)	718 (84)	7.9 (1.3, 321.7)	0.01	29 (100)	14 (93)	16 (100)	2 (100)
Rash ^†^	3 (7)	27 (3)	2.2 (0.4, 7.7)	0.18	2 (7)	1 (7)	1 (6)	0 (0)
Conjunctivitis ^†^	2 (5)	9 (1)	4.4 (0.5, 22.4)	0.10	1 (3)	1 (7)	0 (0)	0 (0)
Arthralgia	14 (32)	119 (14)	2.9 (1.5, 5.6)	**0.002**	7 (24)	8 (53)	4 (25)	1 (50)
Headache	26 (59)	339 (40)	2.2 (1.2, 4.0)	0.01	15 (52)	12 (80)	7 (44)	2 (100)
Retroorbital pain	15 (34)	64 (8)	6.4 (3.2, 12.5)	**<0.001**	11 (38)	6 (40)	5 (31)	1 (50)
Muscle pain/aches	16 (36)	141 (17)	2.9 (1.5, 5.5)	**0.001**	8 (28)	7 (47)	6 (38)	2 (100)
Chills	19 (43)	139 (16)	3.9 (2.1, 7.3)	**<0.001**	11 (38)	8 (53)	7 (44)	2 (100)
Jaundice ^†^	1 (2)	4 (0.5)	4.9 (0.1, 50.9)	0.22	1 (3)	0 (0)	0 (0)	0 (0)
Night or unusual sweats ^†^	4 (9)	26 (3)	3.2 (0.8, 9.8)	0.05	4 (14)	2 (13)	0 (0)	0 (0)
Dysuria ^†^	3 (7)	8 (1)	7.7 (1.3, 33.5)	0.01	3 (10)	0 (0)	0 (0)	0 (0)
Loss of appetite ^†^	3 (7)	4 (0.5)	15.5 (2.2, 93.9)	**0.003**	2 (7)	1 (7)	0 (0)	0 (0)
Fatigue ^†^	2 (5)	10 (1)	4.0 (0.4, 19.6)	0.11	2 (7)	0 (0)	0 (0)	0 (0)
Respiratory								
Cough	17 (39)	472 (56)	0.5 (0.3, 0.9)	0.03	9 (31)	6 (40)	6 (38)	2 (100)
Sore throat	11 (25)	279 (33)	0.7 (0.3, 1.4)	0.28	6 (21)	4 (27)	4 (25)	1 (50)
Wheezing ^†^	1 (2)	42 (5)	0.4 (0, 2.8)	0.72	1 (3)	0 (0)	0 (0)	0 (0)
Chest pain ^†^	2 (5)	95 (11)	0.4 (0, 1.5)	0.22	2 (7)	0 (0)	1 (6)	1 (50)
Sneezing ^†^	2 (5)	150 (18)	0.2 (0, 0.9)	0.02	2 (7)	0 (0)	1 (6)	0 (0)
Difficulty breathing	7 (16)	174 (20)	0.7 (0.3, 1.7)	0.46	4 (14)	1 (7)	2 (13)	1 (50)
Coryza/congestion/rhinorrhea	10 (23)	309 (36)	0.5 (0.3, 1.1)	0.07	8 (28)	1 (7)	6 (38)	2 (100)
Gastrointestinal								
Diarrhea	8 (18)	164 (19)	0.9 (0.4, 2.0)	0.86	6 (21)	2 (13)	3 (19)	1 (50)
Nausea	8 (18)	117 (14)	1.4 (0.6, 3.1)	0.41	6 (21)	2 (13)	2 (13)	1 (50)
Vomiting	10 (23)	156 (18)	1.3 (0.6, 2.7)	0.47	8 (28)	2 (13)	4 (25)	1 (50)
Abdominal/stomach pain	7 (16)	126 (15)	1.1 (0.5, 2.5)	0.84	6 (21)	1 (7)	2 (13)	1 (50)
Upset stomach ^†^	5 (11)	40 (5)	2.6 (0.8, 7.1)	0.06	3 (10)	2 (13)	1 (6)	1 (50)

^†^ Fisher’s exact *p*-value and confidence interval reported. * *p*-value for significance set at 0.002 using the Bonferroni correction to adjust for multiple comparisons. *p*-values in bold were entered into multivariable model.

**Table 6:** Clinical findings at presentation and outcome of DENV-positive cases.

Clinical Outcome	DENV Positive *n* = 44 *n* (%)	DENV-1 *n* = 29 *n* (%)	DENV-2 *n* = 15 *n* (%)	DENV-3 *n* = 15 *n* (%)	DENV-4 *n* = 2 *n* (%)	Monoinfection *n* = 27 *n* (%)	Coinfection *n* = 17 *n* (%)
Elevated hematocrit (male > 54%, female > 48%)	0 (0)	0 (0)	0 (0)	0 (0)	0 (0)	0 (0)	0 (0)
Thrombocytopenia (platelets < 150 × 10^3^/μL)	10 (23)	5 (17)	5 (33)	2 (13)	0 (0)	8 (30)	2 (12)
Leukopenia (<5000/μL)	10 (23)	5 (17)	5 (33)	2 (13)	0 (0)	8 (30)	2 (12)
Leukocytosis (>10,000/μL)	1 (2)	1 (4)	0 (0)	1 (6)	1 (50)	0 (0)	1 (6)
Elevated AST (>40 U/L)	4 (9)	1 (4)	3 (20)	0 (0)	0 (0)	4 (15)	0 (0)
Elevated ALT (>40 U/L)	3 (7)	1 (4)	2 (13)	0 (0)	0 (0)	3 (11)	0 (0)
Hospital admission	2 (5)	0 (0)	2 (13)	0 (0)	0 (0)	2 (7)	0 (0)
Death Met WHO suggested case definition ^†^	0 (0) 25 (57)	0 (0) 15 (52)	0 (0) 11 (73)	0 (0) 8 (53)	0 (0) 2 (100)	0 (0) 15 (56)	0 (0) 10 (59)

AST: aspartate transaminase. ALT: alanine transaminase. ^†^ Reported fever and two or more of the following signs and symptoms: nausea, vomiting, rash, arthralgia, headache, retroorbital pain, muscle pain, chest pain, leukopenia, abdominal/stomach pain, fatigue, elevated hematocrit with thrombocytopenia.

## Data Availability

The data presented in this study are available on request from the corresponding author. The data are not publicly available due to confidentiality of research participants.
